# Supplementary material for: KIF1A-mediated trafficking is required for neuronal autophagy in human neurons
Source: bioRxiv. 2026 Jul 24:2026.07.22.740140. Preprint. [Version 1] doi: 10.64898/2026.07.22.740140 (PMC13419745; doi:10.64898/2026.07.22.740140)
Supplement: 1 [file NIHPP2026.07.22.740140v1-supplement-1.pdf]

bioRxiv preprint doi: https://doi.org/10.64898/2026.07.22.740140; this version posted July 24, 2026. The copyright holder for this preprint (which was not certified by peer review) is the author/funder, who has granted bioRxiv a license to display the preprint in perpetuity. It is made available under a CC-BY 4.0 International license.

Supplemental Figure 1

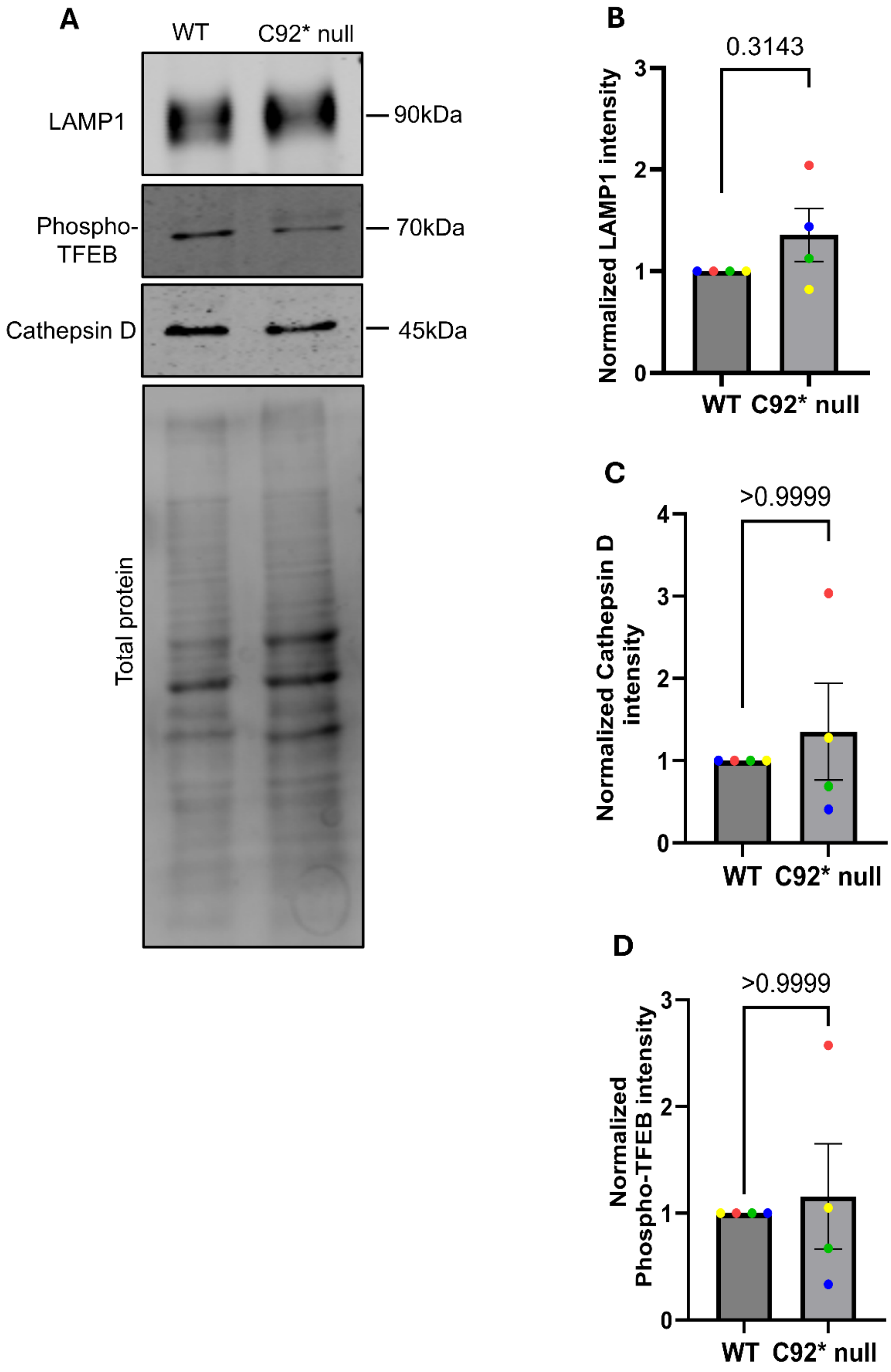

bioRxiv preprint doi: https://doi.org/10.64898/2026.07.22.740140; this version posted July 24, 2026. The copyright holder for this preprint (which was not certified by peer review) is the author/funder, who has granted bioRxiv a license to display the preprint in perpetuity. It is made available under a CC-BY 4.0 International license.

Supplemental figure 1: Lysosome protein expression in WT and C92* null neurons.

A) Representative western blot image probed for LAMP1, phospo-TFEB and cathepsin D in WT and C92* null neurons. Blot was stripped each time when probing for a different protein.

B) Bar graph showing LAMP-1 intensity normalized to total protein of WT and C92* null neurons. Graph shows mean ± standard deviation of experimental replicates (N of 4). Each color represents one replicate. P-values determined by Mann Whitney test.

C) Bar graph showing cathepsin D intensity normalized to total protein of WT and C92* null neurons. Graph shows mean ± standard deviation of experimental replicates (N of 4). Each color represents one replicate. P-values determined by Mann Whitney test.

D) Bar graph showing phospho-TFEB intensity normalized to total protein of WT and C92* null neurons. Graph shows mean ± standard deviation of experimental replicates (N of 4). Each color represents one replicate. P-values determined by Mann Whitney test.

bioRxiv preprint doi: https://doi.org/10.64898/2026.07.22.740140; this version posted July 24, 2026. The copyright holder for this preprint (which was not certified by peer review) is the author/funder, who has granted bioRxiv a license to display the preprint in perpetuity. It is made available under a CC-BY 4.0 International license.

Supplemental Figure 2

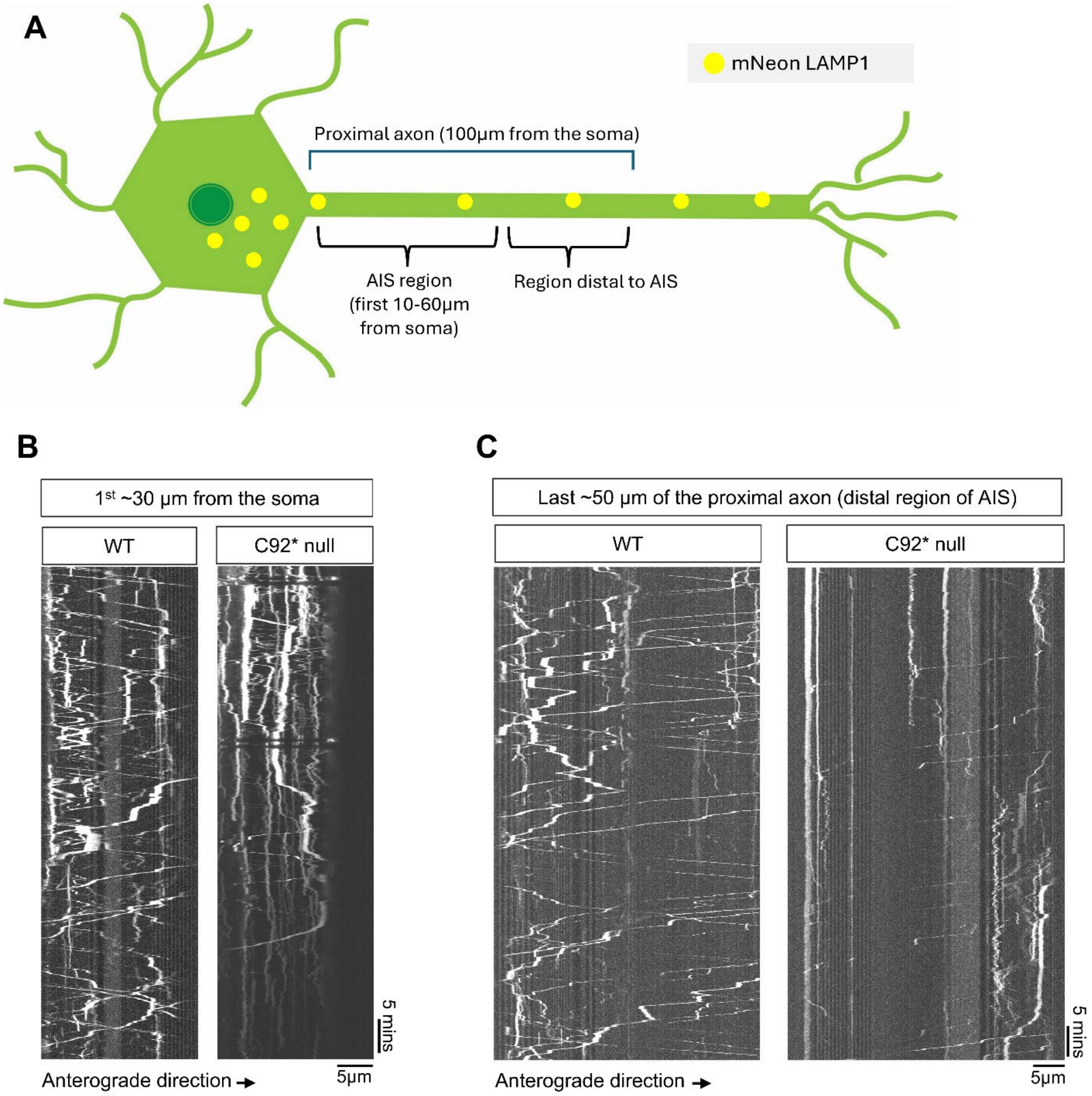

bioRxiv preprint doi: https://doi.org/10.64898/2026.07.22.740140; this version posted July 24, 2026. The copyright holder for this preprint (which was not certified by peer review) is the author/funder, who has granted bioRxiv a license to display the preprint in perpetuity. It is made available under a CC-BY 4.0 International license.

Supplemental figure 2: Lysosome trafficking at the proximal axon in WT and C92* null neurons.

A) Schematic of experiment. WT and C92* null neurons were transfected with mNeon LAMP1. Videos were recorded within the AIS region (10-60μm from the soma) and after (distal to the AIS immediately following the 60μm mark). Proximal region defined as 100μm from the soma.

B) WT Kymograph (**generated from Supplemental Video 1**) and C92* null kymograph (**generated from Supplemental Video 2**) showing LAMP1 tracks within the region of the proximal axon labeled as the AIS. 5FPS videos collected and imaged for 5 minutes. Scale bar 5μm.

C) WT Kymograph (**generated from Supplemental Video 3**) and C92* null kymograph (**generated from Supplemental Video 4)** showing LAMP1 tracks from the same neuron (**Supplemental Video 1, Supplemental Video 2**, **Supplemental figure 2B**) but within the region of the proximal axon labeled as the region distal to the AIS. 5FPS videos collected and imaged for 5 minutes. Scale bar 5μm.

bioRxiv preprint doi: https://doi.org/10.64898/2026.07.22.740140; this version posted July 24, 2026. The copyright holder for this preprint (which was not certified by peer review) is the author/funder, who has granted bioRxiv a license to display the preprint in perpetuity. It is made available under a CC-BY 4.0 International license.

Supplemental Figure 3

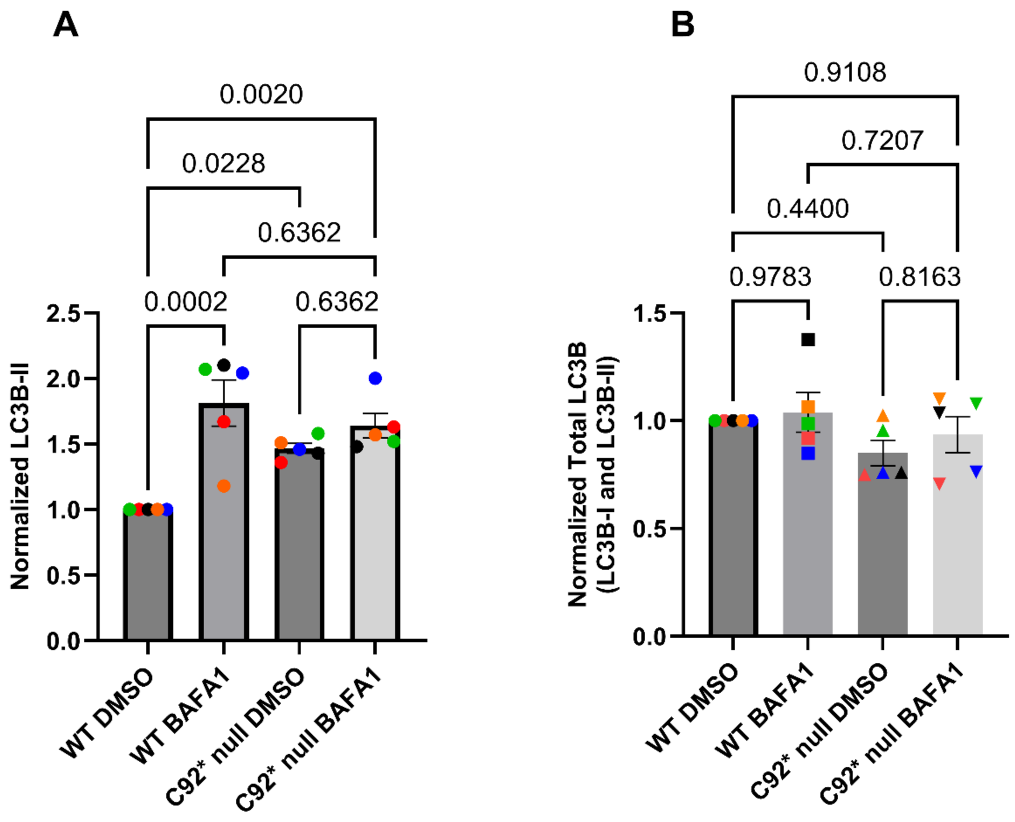

bioRxiv preprint doi: https://doi.org/10.64898/2026.07.22.740140; this version posted July 24, 2026. The copyright holder for this preprint (which was not certified by peer review) is the author/funder, who has granted bioRxiv a license to display the preprint in perpetuity. It is made available under a CC-BY 4.0 International license.

Supplemental figure 3: LC3B-II and total LC3B levels in WT and C92* neurons.

A) Bar graph showing the western blot levels of LC3B-II normalized to total protein in WT and C92* neurons treated with DMSO or BafA1 (see figure 7B for western blot). Graph shows mean ± standard deviation of experimental replicates Each color represents one replicate. P-values determined by Ordinary One-way ANOVA with Tukey's multiple comparison's test.

B) Bar graph showing the western blot levels of total LC3B (LC3B-I and LC3B-II) normalized to total protein in WT and C92* neurons treated with DMSO or BafA1 (see figure 7B for western blot). Graph shows mean ± standard deviation of experimental replicates (N of 5). Each color represents one replicate. P-values determined by Ordinary One-way ANOVA with Tukey's multiple comparison's test.

bioRxiv preprint doi: https://doi.org/10.64898/2026.07.22.740140; this version posted July 24, 2026. The copyright holder for this preprint (which was not certified by peer review) is the author/funder, who has granted bioRxiv a license to display the preprint in perpetuity. It is made available under a CC-BY 4.0 International license.

Supplemental Figure 4

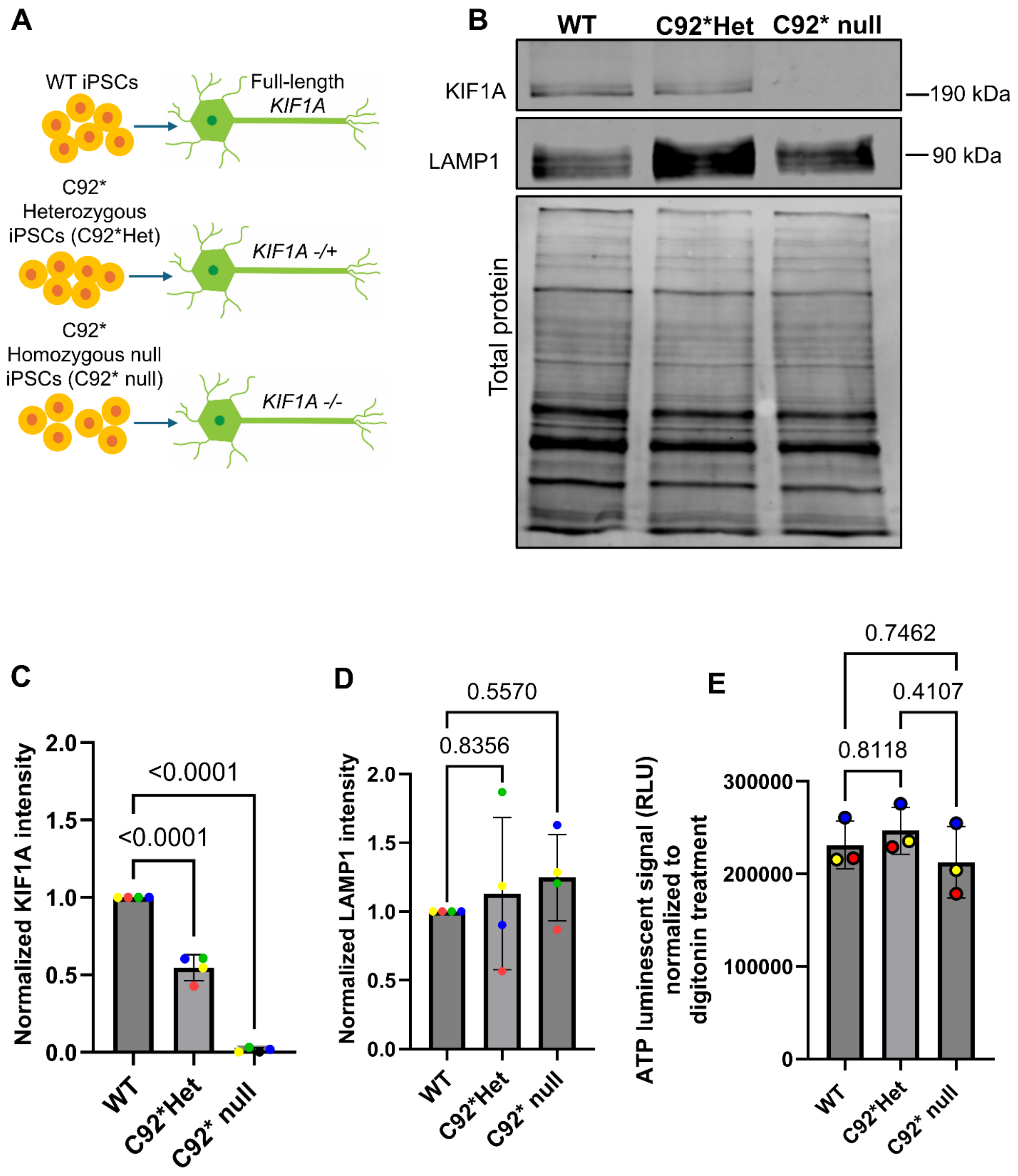

bioRxiv preprint doi: https://doi.org/10.64898/2026.07.22.740140; this version posted July 24, 2026. The copyright holder for this preprint (which was not certified by peer review) is the author/funder, who has granted bioRxiv a license to display the preprint in perpetuity. It is made available under a CC-BY 4.0 International license.

Supplemental figure 4: WT, C92*Het, C92* null KIF1A and LAMP1 protein expression.

A) WT iPSCs and iPSCs gene-edited to endogenously express the C92* heterozygous truncating variant (C92*Het) and C92* null variant were differentiated to cortical-like glutamatergic neurons.

B) Representative western blot showing KIF1A (190kDa) and LAMP1 (90kDa) protein expression for DIV21 WT, C92*Het and C92* null neurons.

C) Quantification of relative protein levels of KIF1A DIV21 neuron lysate, corresponding to example western blot displayed in Supplemental figure 1A. Graph shows mean ± standard deviation; n = 4 independent experiments. Each color represents 1 replicate. WT average =1.00, C92*Het average = 0.55, C92* null average= 0.01. Reported *p*-values are from Ordinary one-way ANOVA with Dunnett's multiple comparisons.

D) Quantification of relative protein levels of LAMP-1 in DIV21 neuron lysate, corresponding to example western blot displayed in in Supplemental figure 1A. Graph shows mean ± standard deviation; N = 4 independent experiments. Each color represents 1 replicate. WT average =1.00, C92*Het average = 1.13, C92* null average= 1.23. Reported *p*-values are from Ordinary one-way ANOVA with Dunnett's multiple comparisons.

E) ATP assay on WT, C92*Het and C92* null with Mitochondrial ToxGlo™ Assay kit. DIV21 neurons were cultured in 96-well plates and treated with an ATP detection reagent, resulting in cell lysis and generation of a luminescent signal (RLU) proportional to the amount of ATP present. Luminescent signal normalized to digitonin treated condition. N of 3 biological replicates. WT average= 231213 RLU, C92*Het average= 246864RLU, C92* null average= 212533 RLU. P-values determined by Ordinary one-way ANOVA with Tukey's Multiple comparison.
